# Methylation analysis for postpartum depression: a case control study

**DOI:** 10.1186/s12888-019-2172-x

**Published:** 2019-06-20

**Authors:** Yukako Nakamura, Masahiro Nakatochi, Shohko Kunimoto, Takashi Okada, Branko Aleksic, Miho Toyama, Tomoko Shiino, Mako Morikawa, Aya Yamauchi, Akira Yoshimi, Yoko Furukawa-Hibi, Taku Nagai, Masako Ohara, Chika Kubota, Kiyofumi Yamada, Masahiko Ando, Norio Ozaki

**Affiliations:** 1 Department of Psychiatry, Nagoya University Graduate School of Medicine, 65 Tsurumai-cho, Showa-ku, Nagoya, Aichi 466-8550 Japan; 2 Data Coordinating Center, Department of Advanced Medicine, Nagoya University Hospital, 65 Tsurumai-cho, Showa-ku, Nagoya, Aichi 466-8550 Japan; 3 Division of Clinical Sciences and Neuropsychopharmacology, Faculty and Graduate School of Pharmacy, Meijo University, 1-501 Shiogamaguchi, Tempaku-ku, Nagoya, Aichi 468-8503 Japan; 4 Department of Experimental and Clinical Pharmacy, Nagoya City University Graduate School of Pharmaceutical Sciences, Tanabe-dori, Mizuhoku, Nagoya, Aichi 467-8603 Japan; 5 Department of Neuropsychopharmacology and Hospital Pharmacy, Nagoya University Graduate School of Medicine, 65 Tsurumai-cho, Showa-ku, Nagoya, Aichi 466-8560 Japan; 6 Department of Nursing, Nagoya University Graduate School of Medicine, 1-1-20 Daiko-Minami, Higashi-ku, Nagoya, Aichi 461-8673 Japan

**Keywords:** DNA methylation, Postpartum depression, Epigenome-wide association study, Edinburgh postnatal depression scale (EPDS)

## Abstract

**Background:**

Postpartum depression (PPD) is a major depressive disorder that occurs after childbirth. Objective diagnostic and predictive methods for PPD are important for early detection and appropriate intervention. DNA methylation has been recognized as a potential biomarker for major depressive disorder. In this study, we used methylation analysis and peripheral blood to search for biomarkers that could to lead to the development a predictive method for PPD.

**Methods:**

Study participants included 36 pregnant women (18 cases and 18 controls determined after childbirth). Genome-wide DNA methylation profiles were obtained by analysis with an Infinium Human Methylation 450BeadChip. The association of DNA methylation status at each DNA methylation site with PPD was assessed using linear regression analysis. We also conducted functional enrichment analysis of PPD using The Database for Annotation, Visualization and Integrated Discovery 6.8 to explore enriched functional-related gene groups for PPD.

**Results:**

In the analysis with postpartum depressed state as an independent variable, the difference in methylation frequency between the postpartum non-depressed group and the postpartum depressed group was small, and sites with genome-wide significant differences were not confirmed. After analysis by The Database for Annotation, Visualization and Integrated Discovery 6.8, we revealed four gene ontology terms, including axon guidance, related to postpartum depression.

**Conclusions:**

These findings may help with the development of an objective predictive method for PPD.

**Electronic supplementary material:**

The online version of this article (10.1186/s12888-019-2172-x) contains supplementary material, which is available to authorized users.

## Background

Postpartum depression (PPD) is a type of major depressive disorder that occurs after childbirth; the prevalence of PPD is estimated at approximately 13% [1]. Our study showed that 10.4% of postpartum women in Japan experienced depressive symptomatology based on the Edinburgh Postnatal Depression Scale (EPDS) [2]. PPD is related to maternal suicide [3] and affects children’s development [4]. One study reported that among women who had no history of psychological disorder but who were diagnosed with PPD, 54% had bipolar disorder [5]. Therefore, objective diagnostic and predictive methods for PPD are important for early detection of patients and appropriate intervention. The Edinburgh Postnatal Depression Scale (EPDS) is the most widely used screening questionnaire for PPD [6]. However, the EPDS is only a screening instrument and cannot be used to make a clinical diagnosis [7]. A diagnostic method for depression using Near-Infrared Spectroscopy was created [8], but sufficient sensitivity and specificity were not shown. Therefore, a new index is required. It is important to search for biomarkers that are predictive indicators of PPD for early detection and intervention.

Major depressive disorder is associated with both genetic and environmental factors and is considered to be associated with an epigenetic mechanism [9]. DNA methylation is the most widely studied epigenetic mechanism; it reveals changes in gene expression that are not due to alterations in the DNA sequence [10]. DNA methylation within gene promoters generally exerts a repressive effect on gene transcription, and use of this technique has been considered a potential way to identify possible biomarkers for depressive disorder [9].

Recent advances in epigenome-wide analyses have extended the field beyond a gene candidate approach toward a more comprehensive epigenome approach [11]. In terms of epigenome-wide analyses of PPD, Kaminsky et al. reported estrogen receptor- and oxytocin receptor-mediated epigenetic changes associated with PPD [12–14]. An association between PPD and the oxytocin receptor gene has also been reported with methylation analysis using the candidate gene approach [15].

In a methylation study, it is necessary to consider variations in methylation patterns due to racial and ethnic differences [16]. Therefore, a methylation analysis that examines Japanese women is indispensable for the development of PPD predictive methods in Japan. The results of this study from a methylation analysis may clarify the characteristics of the methylation frequency of women who exhibit PPD and lead to the development of an effective predictive method for PPD in Japanese women.

The pathogenic processes for psychiatric disorders likely involve the histologic pathology of the brain. However, brain tissue is not readily accessible in living patients, so biomarker studies typically use blood [17]. An association between methylation frequencies of the brain and peripheral blood has been reported [18, 19]. Similarly, a relatively high correlation has been observed between gene expression in the brain and that in peripheral blood [20]. Furthermore, a methylome-wide association study reported that peripheral blood can be a biomarker for schizophrenia [17]. Therefore, it is suggested that the methylation frequency of peripheral blood may be a useful biomarker for PPD diagnosis. Based on the above, we used methylation analysis and peripheral blood to search for biomarkers that could lead to the development of a predictive method for PPD.

## Methods

### Participants

Women were recruited from Nagoya University Hospital and its associated institutes. Inclusion criteria were participation in perinatal classes for pregnant Japanese women before week 25 of pregnancy**,** age ≥ 20 years, and the ability to understand and speak Japanese. The study was explained both orally and in writing to all participants during the perinatal classes, and all participants provided written informed consent**.**

### Procedure

Participants were provided with a consent form and a self-reporting questionnaire during a perinatal class. The questionnaire included the EPDS and demographic questions, such as age, parity and current and previous mental illness (Additional file 1). Women were asked to complete the questionnaire in early pregnancy (before week 25) and return their responses, along with the agreement document, via mail using the provided self-addressed envelope. We provided self-addressed envelopes and postage on these envelopes. Questionnaires were mailed to women again around week 36 of pregnancy and 1 month after delivery, and returned in the same manner.

### EPDS

The EPDS is a 10-item self-reporting questionnaire that assesses PPD. Each item is scored on a 4-point Likert scale, with higher scores indicating worse depression [21]. The EPDS was translated into Japanese and then reverse translated back into English, and was determined to be the same as the English version [22]. The Japanese version of the EPDS showed good internal consistency (Cronbach’s alpha = 0.78) and test-retest reliability (Spearman’s correlation coefficient = 0.92) [22]. In the original version, the cut-off point is 12/13 [21], and the EPDS cut-off point 12/13 is recommended for English-speaking women [23]. In the non-English version, a cut-off point lower than 12/13 has been reported to be appropriate [24–28], as cut-off point requires validation for specific populations [29]. Although one report regarding the EPDS recommended the cut-off point 12/13 in the Japanese version [30], the cut-off point in that study was calculated by subjects who excluded pregnant women with an EPDS ≦8 during pregnancy. Participant in this study is different from that study [30]. For example, we included women with an EPDS ≦ 8 during pregnancy. As a result, we used a cut-off point 8/9 in Japanese version of the EPDS by Okano et al. [22]. A score ≥ 9 on the Japanese version of the EPDS has been used as an indication of major depressive episodes. The test has a sensitivity of 75% and a specificity of 93% [22]. We used the 8/9 cut-off point and defined a score ≥ 9 as depressive episodes.

### Definition of PPD

We evaluated depressive symptoms using the EPDS during early pregnancy (before week 25), late pregnancy (around week 36), and 1 month after birth. We measured EPDS scores twice in pregnancy, to confirm that participants were not depressed in pregnancy both cases and controls. The incidence of peak depression in the postnatal period has been debated [31],and one report found a peak 6 weeks after birth [32]. On the other hand, “maternity blues” frequently occur during the period immediately after childbirth to 10 days after childbirth [33]. Prevalence rates of maternity blues vary from 40 to 80% [34]. We considered that the time period immediately after childbirth, when many pregnant women experience maternity blues, was not a suitable time to search for PPD biomarkers. Therefore, we chose 1 month after birth as time point for postpartum EPDS and blood collection. We defined cases and controls based on the results of the EPDS scores. The controls scored under the cut-off point before week 25, around week 36, and 1 month after birth. The cases scored under the cut-off point before week 25 and around week 36, but scored over the cut-off point at 1 month postpartum. The control group excluded participants with current or previous mental illness. In addition, we matched cases and controls in terms of age, parity, gestational weeks, and the day of blood collection after birth. A total of 18 cases and 18 controls were identified.

### DNA methylation analysis

We collected peripheral blood from all participants about 1 month after birth for extraction of genomic DNA. Venous blood was collected into tubes containing EDTA, and genomic DNA was extracted from peripheral blood using the Qiagen QIAamp DNA bloods Kit (Qiagen, Hilden, Germany). The extracted DNA was stored at − 30 °C until the start of the experiment. We performed DNA extraction from October 2006 to March 2013, and conducted experiments from March 2013 to November 2015.

Genomic DNA was extracted from peripheral blood. Bisulfite conversion of genomic DNA from peripheral blood was done using the EpiTect Plus Bisulfite Kits (Qiagen Ltd., Hilden, Germany). Genome-wide DNA methylation profiles were obtained using the Infinium Human Methylation 450BeadChip according to the manufacturer’s instructions (Illumina, San Diego, CA, USA). To minimize potential batch effects, cases and controls were processed together and run on the same BeadChip. Samples were randomly positioned on each BeadChip to reduce any possible position effects within chips. We used a correction method applied in a previous study to reduce technical bias in the DNA methylation array data [35]. Briefly, DNA methylation values were corrected for background and normalized with the Subset-quantile Within Array Normalization function [36] of minfi in R to adjust for color bias [37]. The minfi is a flexible and comprehensive R package for the analysis of Infinium DNA methylation microarrays, and R is an open-source, free software program for statistical analysis.

DNA methylation level was quantified as an M value, which can be converted to a β value according to the equation: β = 2^M^/(2^M^ + 1) [38]. The M value for all DNA methylation sites was corrected for batch effects with the ComBat function of sva in R. sva is an R package that contains functions for removing batch effects [39] and the ComBat function adjusts for known batches using an empirical Bayesian framework [40].

We estimated the cell type composition for each sample with the estimate Cell Counts function [41] of minfi in R. For each sample, probes with a detection *p* value ≥0.05 were assigned a status of “not detected”; this led to the exclusion of 1316 probes (5% of samples). We also excluded 30,819 probes previously found to be cross-reactive (≥47 bases) [42] and probes on the Y chromosome. Probes containing single nucleotide polymorphisms have been found to influence the assessment of DNA methylation status when using the Infinium Human Methylation 450BeadChip [43]. We therefore filtered out probes that contained single nucleotide polymorphisms with a minor allele frequency > 0.01 based on 1000 Genomes project samples of ASN (ASN: Han Chinese individuals in Beijing, Southern Han Chinese, and Japanese in Tokyo) [42] to reduce the frequency of false positives. Finally, a total of 379,277 DNA methylation sites remained.

### Statistical analysis

The association of PPD with DNA methylation status at each DNA methylation site was assessed using a linear regression analysis, with DNA methylation status at each site as a dependent variable, and PPD label (case = 1, control = 0) and covariates as independent variables. The covariates comprised age and the cell type composition of samples. All *p* values were corrected for genomic control. The significance level α for the epigenome-wide association study analysis was determined by dividing 0.05 by the number of DNA methylation sites for Bonferroni correction (α = 0.05/379,277 = 1.32 × 10^− 7^). A *p* value < 0.05 was considered nominally significant.

Gene ontology (GO) terms are used to describe 3 categories of gene products: biological process, molecular function, and cellular component [44]. These terms are widely used in gene function studies. Identifying GO terms that are overpresented within a given list of genes can increase the understanding of the functional relevance of these genes [45].

For genes that showed differential DNA methylation between cases and controls, gene set enrichment analysis was used to identify enriched GO terms. Analyses were done using the Database for Annotation, Visualization and Integrated Discovery (DAVID) 6.8 [46, 47]. DAVID is a free, online bioinformatics resource that lists a comprehensive set of functional annotations that can be used to identify the biological significance of a list of genes. We selected 1000 sites with the lowest *p* values. A total of 711 genes that were annotated within these 1000 sites were analyzed using DAVID. GO terms with a false discovery rate < 0.05, as calculated by the Benjamini-Hochberg adjustment method, were considered significant.

### Data availability

The datasets used and analyzed during the current study are available from the corresponding author on reasonable request.

## Results

### Characteristics of study subjects

Table 1 shows characteristics of the study subjects. The mean ages of cases and controls were 33.3 ± 4.4 and 34.6 ± 4.6 years, respectively. There were no significant differences in age, parity, gestational weeks,baby’s weight (g), and blood collection day after birth (days) between cases and controls. EPDS score at 1 month after birth was significantly higher among cases compared with controls. The range of EPDS scores at early pregnancy and late pregnancy was 0–8 for cases and controls. The range of EPDS scores at 1 month after birth was 9–25 for cases and 0–8 for controls.

**Table 1:** Characteristics of study subjects

	Controls (*n* = 18) Mean (SD)		Cases (*n* = 18) Mean (SD)		*p* value
Age (years)	34.6	(4.6)	33.3	(4.4)	0.386
Parity	1.2	(0.4)	1.1	(0.3)	0.594
Gestational weeks	39.1	(1.3)	38.9	(1.0)	0.480
Baby’s weight (g)	3035	(304.7)	2993	(237.9)	0.684
Blood collection day after birth (days)	30.1	(7.6)	26.0	(10.7)	0.195
EPDS score 1 month after birth	2.3	(2.4)	13.6	(4.2)	1.9 × 10^− 11^

*EPDS* Edinburgh Postnatal Depression Scale

### Association analysis for DNA methylation status

**Fig. 1:**
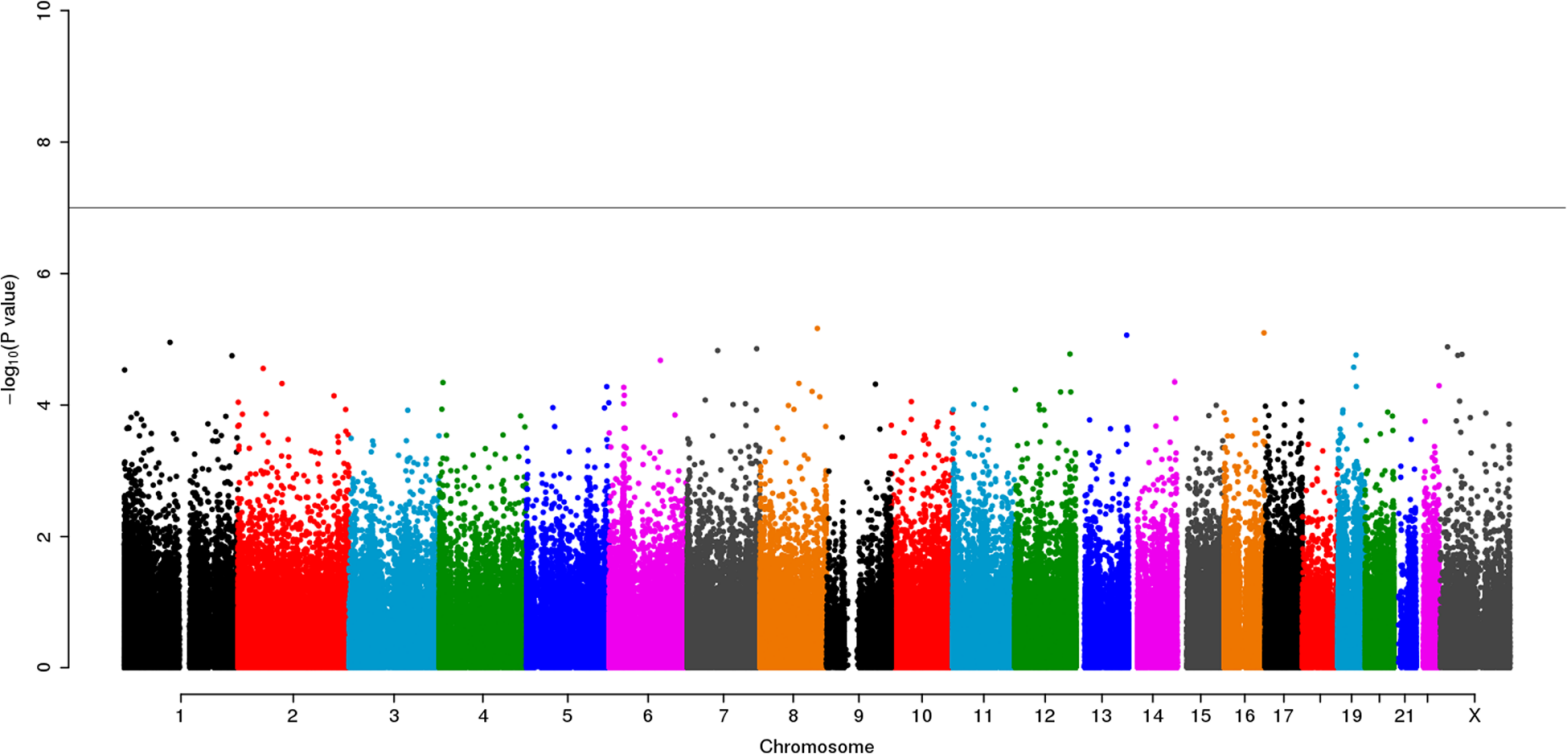
A Manhattan plot for the epigenome-wide association study analysis of PPD and DNA methylation. The genome-wide significance level (α = 1.32 × 10^− 7^) is denoted by the horizontal line. The *p* values were corrected for genomic control

**Fig. 2:**
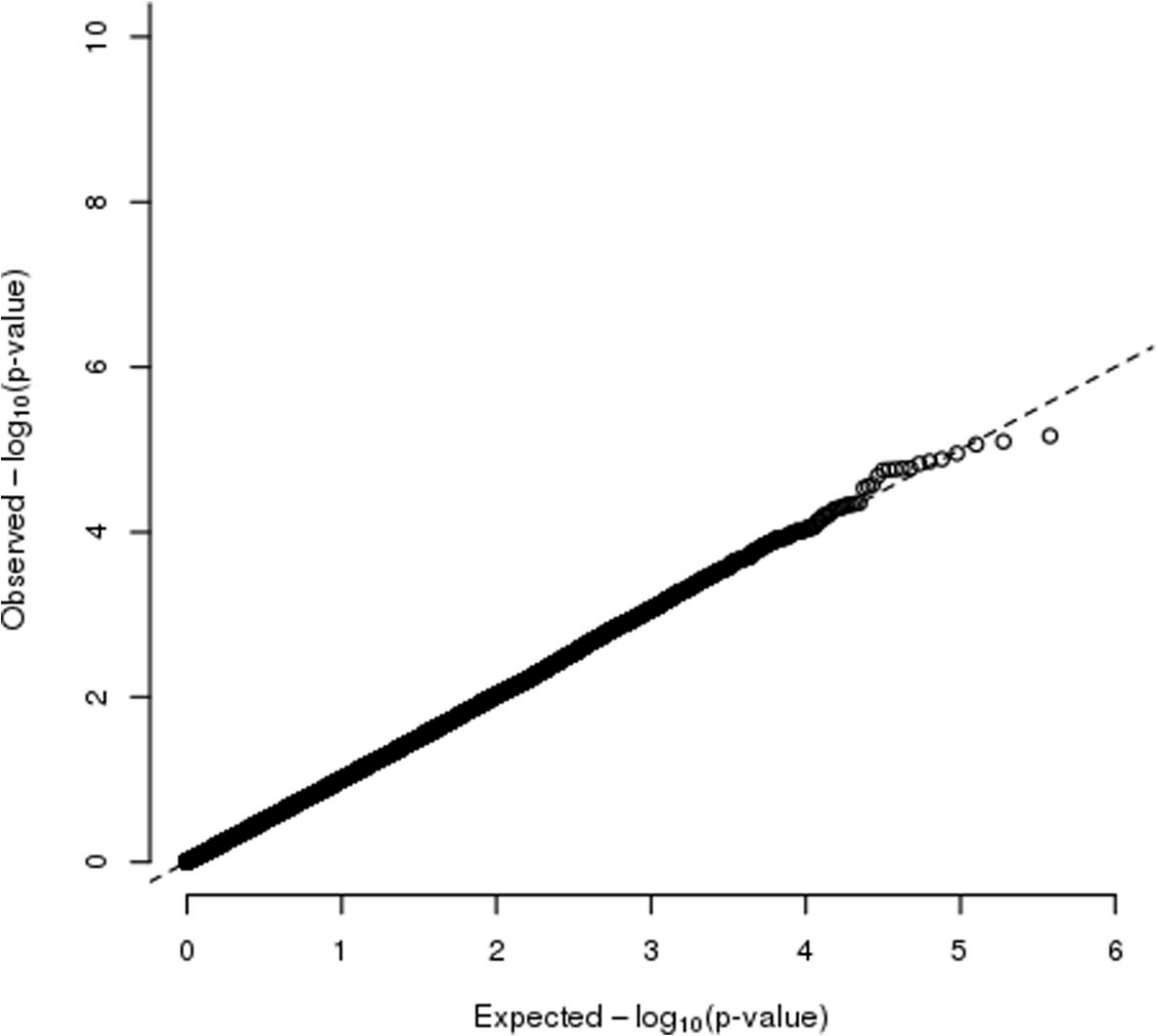
A quantile-quantile plot of the expected versus the observed –log_10_(*p* value) for tests of association between PPD and DNA methylation sites. The *p* values were corrected for genomic control

After initial processing, 18 cases and 18 controls as well as 379,277 DNA methylation sites remained for subsequent analysis. We performed an association analysis for DNA methylation level and PPD and found no genome-wide significant association (Fig. 1). In addition, no outliers were detected in the quantile-quantile plot of −log_10_(p) for the 379,277 tests of association between DNA methylation status and cases-controls (Fig. 2).

As a result of DAVID 6.8 analysis of the 711 genes associated with the 1000 sites with the lowest *p* values (Additional file 2: Table S1), we found four GOTERM DIRECT terms and 16 GOTERM FAT terms with a false discovery rate < 0.05 (Table 2). GOTERM DIRECT provides GO mappings directly annotated by the source database and GOTERM FAT filters out very broad GO terms based on a measured specificity of each term. We showed 22 genes of the axon guidance in which the Fold Enrichment was largest among the four GOTERM DIRECT terms (Table 3).

**Table 2:** Gene ontology term enrichment analysis with DAVID

Category	Term	Count	Fold Enrichment	FDR^a^
GOTERM_BP_DIRECT	GO:0007411~axon guidance	22	3.27	0.0088
GOTERM_BP_FAT	GO:0097485~neuron projection guidance	27	2.86	0.0074
GOTERM_BP_FAT	GO:0007411~axon guidance	27	2.88	0.0136
GOTERM_BP_FAT	GO:0030182~neuron differentiation	83	1.61	0.0235
GOTERM_BP_FAT	GO:0032990~cell part morphogenesis	59	1.73	0.0257
GOTERM_BP_FAT	GO:0007409~axonogenesis	37	2.13	0.0259
GOTERM_BP_FAT	GO:0048699~generation of neurons	89	1.56	0.0271
GOTERM_BP_FAT	GO:0048667~cell morphogenesis involved in neuron differentiation	42	2.02	0.0274
GOTERM_BP_FAT	GO:0061564~axon development	38	2.04	0.0278
GOTERM_BP_FAT	GO:0048666~neuron development	67	1.66	0.0284
GOTERM_BP_FAT	GO:0021953~central nervous system neuron differentiation	21	2.89	0.0305
GOTERM_BP_FAT	GO:0048858~cell projection morphogenesis	58	1.74	0.0305
GOTERM_BP_FAT	GO:0048812~neuron projection morphogenesis	43	1.89	0.0389
GOTERM_BP_FAT	GO:0031175~neuron projection development	58	1.70	0.0409
GOTERM_BP_FAT	GO:0022008~neurogenesis	90	1.48	0.0461
GOTERM_BP_FAT	GO:0035556~intracellular signal transduction	147	1.33	0.0486
GOTERM_CC_DIRECT	GO:0005654~nucleoplasm	157	1.38	0.0040
GOTERM_MF_DIRECT	GO:0005515~protein binding	421	1.15	0.0032
GOTERM_MF_DIRECT	GO:0003714~transcription corepressor activity	24	2.91	0.0033
GOTERM_MF_FAT	GO:0003714~transcription corepressor activity	24	2.63	0.0495

*DAVID* The Database for Annotation, Visualization and Integrated Discovery 6.8, *BP* biological process, *CC* cellular component, *MF* molecular function, *FDR* false discovery rate

^a^ Calculated by the Benjamini-Hochberg adjustment method

**Table 3:** Gene list of axon guidance using DAVID

Category	Term	Genes
GOTERM_BP_DIRECT	GO:0007411 ~axon guidance	kinesin family member 26A(KIF26A), ephrin A2 (EFNA2), neurexin 1 (NRXN1), glypican 1 (GPC1), dorsal inhibitory axon guidance protein (DRAXIN), growth factor receptor bound protein 2 (GRB2), isoprenoid synthase domain containing (ISPD), FYN proto-oncogene, Src family tyrosine kinase (FYN), ribosomal protein S6 kinase A5 (RPS6KA5), mitogen-activated protein kinase 8 interacting protein 3 (MAPK8IP3), nerve growth factor receptor (NGFR), laminin subunit alpha 2 (LAMA2), EPH receptor A8 (EPHA8), colony stimulating factor 1 receptor (CSF1R), sodium voltage-gated channel beta subunit 1 (SCN1B), EPH receptor B1 (EPHB1), contactin 2 (CNTN2), ubiquitin specific peptidase 33 (USP33), cadherin 4 (CDH4), unc-5 netrin receptor A (UNC5A), growth differentiation factor 7 (GDF7), Wnt family member 5A (WNT5A)

*DAVID* The Database for Annotation, Visualization and Integrated Discovery 6.8

## Discussion

In this study, we used methylation analysis and peripheral blood to search for biomarkers that could lead to development of an objective predictive method for PPD.

In the analysis with postpartum depressed state as an independent variable, the difference in methylation frequency between the non-depressed group and the postpartum depressed group was small, and sites with a genome-wide significant difference were not confirmed. For that reason, our results could not confirm the relationship between estrogen and oxytocin receptors and PPD as reported by Kaminsky and colleagues [12–14]. It is not clear whether the reason is due to racial differences or other factors. This study had a small sample size (18 controls and 18 cases), and our results are preliminary in nature. Future examination is necessary.

We found 4 GOTERM DIRECT terms: protein binding, nucleoplasm, transcription corepressor activity, and axon guidance. Among them, axon guidance, which had the largest fold enrichment, is an important process to form a neural circuit at the stage of nervous system development, and a relationship with stress [48] and depression [49, 50] has been noted. As a result of epigenome-wide studies of depression, axon guidance was reported as a common disrupted pathway in depression [51]. Among the genes associated with axon guidance, Src family tyrosine kinase (FYN), shown in Table 3, has been reported to be associated with anxiety-related behaviors [52]. In addition, axon guidance pathway has been shown to be associated with neuroticism [53], and neuroticism is a well-known predictive factor for depression [54]. It has been reported that psychological stress may affect brain structure and brain function [55, 56], and may contribute to depression [57]. Our results suggest a relationship between axon guidance and PPD.

### Strengths and limitations

In this study we started recruiting participants before week 25 of pregnancy, so we could collect detailed information from participants and match cases and controls in terms of age, parity, gestational weeks, and the day of blood collection after birth. The association between PPD and age [58, 59], parity [60, 61], and past mental illness [62, 63] has been reported. However, in this study, there were no significant differences in age and parity between the two groups. We excluded participants with current or previous mental illness and who had an EPDS score≧9 during pregnancy. Therefore, it was possible to exclude the influence of age, parity and history with mental illness to depression. Furthermore, there was no significant difference in the blood collection date of peripheral blood used for methylation analysis, and it was possible to compare the methylation status of cases and controls around 30 days postpartum. Additionally, we placed cases and controls on the same chip but in a random position to mitigate a batch effect and minimize technical bias.

This study has several limitations. First, the sample size was small. In this study, we evaluated depressive symptoms between pregnancy and the postpartum period using the EPDS, and performed blood collection after childbirth. When the number of cases is small, a large number of controls is needed to avoid bias. However, it was difficult for us to obtain a large sample. We attempted to overcome the limitation of sample size by appropriate analysis. However, it is possible that the negative findings in this study could be due to the small sample size. It is difficult to make a conclusion based on the analysis of small objects, so results of this study can only be considered preliminary data. Future studies with larger samples are needed. Second, to clarify the difference in postpartum depression, the conditions during pregnancy cases and controls were matched. That is, we excluded participants with current or previous mental illness and who had EPDS score ≥ 9 during pregnancy. Therefore, our study is subject to selection bias and the results do not reflect a general pregnant population. Depression during pregnancy and medical history are considered to be related to PPD, and it is necessary to consider these subjects in the future. Third, we defined cases and controls based on the results of the EPDS scores in this study. However, EPDS is a screening tool as opposed to diagnostic tool, and cases of PPD cannot be determined from EPDS score alone. It was a limitation of this study that diagnostic interviews could not be conducted for all the subjects. Finally, we used peripheral blood as opposed to brain tissue in this study. However, the methylation status of peripheral blood and brain may be different, and although using peripheral blood as a biomarker has a clinical advantage, it is also a study limitation.

## Conclusions

Four GO terms that include sites with differences in methylation intensities between cases and controls were identified. Although epigenetic research in psychiatry is still in its early stages, it may ultimately be useful in the understanding of how temporary or chronic life events, alone or in combination with susceptible genes, may impact neuropsychiatric systems [11]. Although we did not identify any significant genome-wide associations based on DNA methylation sites and PPD, future studies that either confirm or contradict our findings may help with the development of an objective predictive method for PPD.

## Additional files

Additional file 1: Questionnaire. It shows items of the self-reporting questionnaire we used in this study. (PDF 83 kb)

Additional file 2: **Table S1** Result of DAVID 6.8 analysis of the 711 genes associated with the 1000 sites. It shows a result of DAVID 6.8 analysis of the 711 genes associated with the 1000 sites with the lowest *p* values of association analysis. (XLSX 99 kb)

## Data Availability

All the data supporting our findings are contained within the manuscript. Therefore, data sets are not shown.

## Abbreviations

BP: Biological process
CC: Cellular component
DAVID: Database for Annotation, Visualization and Integrated Discovery
EPDS: Edinburgh Postnatal Depression Scale
FDR: False discovery rate
GO: Gene ontology
MF: molecular function
PPD: Postpartum depression
