# Supplementary material for: Methylation analysis for postpartum depression: a case control study
Source: BMC Psychiatry. 2019 Jun 20;19:190. doi: 10.1186/s12888-019-2172-x (PMC6585089; doi:10.1186/s12888-019-2172-x)
Supplement: Supplementary file 1 — Questionnaire. It shows items of the self-reporting questionnaire we used in this study. (PDF 83 kb) [file 12888_2019_2172_MOESM1_ESM.pdf]

Your date of birth: ________________________________

Age: ______________

Estimated date of delivery: _______________________

Your baby's parity: 1st child   2nd child   3rd child   4th child   Other (th child)

Do you have any history of mental illness?

No   Yes (Name of mental illness:)

Baby's date of birth: _______________________________

Birth weight: ___________(g)

Please check the answer that comes closest to how you have felt in the past 7 days, not just how you feel today.

1. I have been able to laugh and see the funny side of things
   - ☐ As much as I always could
   - ☐ Not quite so much now
   - ☐ Definitely not so much now
   - ☐ Not at all

2. I have looked forward with enjoyment to things
   - ☐ As much as I ever did
   - ☐ Rather less than I used to
   - ☐ Definitely less than I used to
   - ☐ Hardly at all

3. I have blamed myself unnecessarily when things went wrong
   - ☐ Yes, most of the time
   - ☐ Yes, some of the time
   - ☐ Not very often
   - ☐ No, never

4. I have been anxious or worried for no good reason
   - ☐ No, not at all
   - ☐ Hardly ever
   - ☐ Yes, sometimes
   - ☐ Yes, very often

5. I have felt scared or panicky for no very good reason
   - ☐ Yes, quite a lot
   - ☐ Yes, sometimes
   - ☐ No, not much
   - ☐ No, not at all

6. Things have been getting on top of me
    - ☐ Yes, most of the time I haven’t been able to cope at all
    - ☐ Yes, sometimes I haven’t been coping as well as usual
    - ☐ No, most of the time I have coped quite well
    - ☐ No, I have been coping as well as ever

7. I have been so unhappy that I have had difficulty sleeping
    - ☐ Yes, most of the time
    - ☐ Yes, sometimes
    - ☐ Not very often
    - ☐ No, not at all

8. I have felt sad or miserable
    - ☐ Yes, most of the time
    - ☐ Yes, quite often
    - ☐ Not very often
    - ☐ No, not at all

9. I have been so unhappy that I have been crying
    - ☐ Yes, most of the time
    - ☐ Yes, quite often
    - ☐ Only occasionally
    - ☐ No, never

10. The thought of harming myself has occurred to me
    - ☐ Yes, quite often
    - ☐ Sometimes
    - ☐ Hardly ever
    - ☐ Never

Date ________________________
